# Plasma Neuron-Specific Enolase is not a reliable biomarker for staging *Trypanosoma brucei rhodesiense* sleeping sickness patients

**DOI:** 10.1186/s13104-022-05981-w

**Published:** 2022-03-07

**Authors:** Charles D. Kato, Dorothy Twesigye, Vincent P. Alibu, Ann Nanteza, Julius Nsubuga, Claire M. Mugasa, Enock Matovu

**Affiliations:** 1 School of Bio-Security, Biotechnical & Laboratory Sciences, College of Veterinary Medicine, Animal Resources & Bio-Security, Makerere University, P.O Box 7062, Kampala, Uganda; 2 College of Natural Sciences, Makerere University, P.O Box 7062, Kampala, Uganda

**Keywords:** Human African trypanosomiasis, Sleeping sickness, Biomarker, Neuron-Specific Enolase

## Abstract

**Objective:**

Currently, the only available staging criterion for *T. b. rhodesiense* requires a lumber puncture to collect and later examine cerebrospinal fluid (CSF). This study examined the potential of plasma Neuron-Specific Enolase (NSE) in discriminating between early and late-stage patients.

**Results:**

When median NSE levels were compared between early and late-stage patients, results showed a significant (P < 0.02) upregulation among late-stage patients (599.8 ng/mL). No significant differences (P > 0.9) in NSE levels were observed between early-stage patients (300 ng/mL) and controls (454 ng/mL). We used Receiver Operator Characteristic (ROC) curves to explore the likelihood of using plasma NSE as a potential stage biomarker in discriminating between early and late-stage HAT patients. Our results showed that NSE demonstrated an area under the curve (AUC) of 0.702 (95% CI 0.583–0.830). A high staging accuracy for NSE was obtained by using a cutoff of > 346.5 ng/mL with a sensitivity of 68.6% (95% CI 55–79.7%) and a specificity of 93.3% (95% CI 70.2–99.7%). Although our results demonstrate that plasma NSE is upregulated in *T. b. rhodesiense* sleeping sickness patients, its value in discriminating between late and early-stage patients is limited. However, future studies could consider improving its specificity by combining it with other identified plasma biomarkers.

**Supplementary Information:**

The online version contains supplementary material available at 10.1186/s13104-022-05981-w.

## Introduction

Human African trypanosomiasis (HAT) also called sleeping sickness is a vector borne neglected tropical disease caused by extracellular protozoan parasites *Trypanosoma brucei rhodesiense* in East and Southern Africa and *T. b. gambiense* in West and Central Africa. Due to sustained control measures, the number of new cases has been on the decline with only 98 *T. b. rhodesiense* new cases reported in 2020 with 90.8% of these found in Malawi [1]. Although the number of reported new cases in endemic regions remains low, the disease is still a public health problem and indeed, due to the zoonotic nature of *T. b. rhodesiense* sleeping sickness, elimination cannot be easily achieved [2].

The pathogenesis of HAT involves two stages. Firstly, the early or haemolymphatic stage characterized by the proliferation of trypanosomes in blood, lymph and other tissues. And later, the late or meningo-encephalitic stage in which parasites invade the central nervous system (CNS). Treatment for *T. b. rhodesiense* HAT still relies on disease stage determination that involves the analysis of cerebrospinal fluid. However, the draw back to this staging criterion is that a lumber puncture is invasive and requires well trained personnel that might not always be available in remote areas [3]. Furthermore, in order to confirm cure, after recovery patients are followed through the examination of cerebrospinal fluid every 6 months for up to 24 months after treatment [2]. However, compliance with this follow-up protocol is usually poor especially among asymptomatic individuals [4] and the requirement for a lumber puncture has been shown to deter suspects from HAT screening and follow-up [5]. Since initial disease diagnosis is done using blood, a blood based staging criteria would be ideal.

Neuron-Specific Enolase (NSE) also known as enolase 2 or γ-enolase a dimeric isoform of the glycolytic enzyme enolase [6] mainly found in neurons has been described as a biomarker for a number of brain disorders due to its elevation in cerebrospinal fluid and blood. Normally, NSE is not secreted into extracellular space, but when neurons are injured, NSE can be leaked into extracellular spaces and subsequently upregulated in CSF and blood. Indeed, in patients with traumatic brain injury, serum NSE levels significantly correlated with injury severity score and CT imaging findings especially among none survivors [7]. During the late stage of HAT, trypanosomes evade the blood–brain barrier to enter and establish within the CNS parenchyma. Studies in experimental animals have demonstrated the presence of astrocytosis and evidence of neuronal loss in some areas of the brain [8, 9], presenting a possibility of NSE linking into extracellular spaces. Because of this observation, a number of studies have investigated the possibility of using NSE in conditions associated with CNS neuronal injury. Mean serum NSE levels have been shown to be significantly upregulated and correlated with the degree of neurological worsening in patients with cerebrovascular stroke [10] and in acute ischemic stroke [11], demonstrating the potential role of NSE as a marker for brain injury. In small cell lung cancer, NSE was reported to be an effective biomarker with a sensitivity of 74%. In HAT, the only study that has evaluated the potential of plasma NSE as a stage diagnostic biomarker reported a 75% sensitivity and a specificity of 72% [12]. However, till now translation of this marker into a useful staging tool has not been done due to limited number of validation studies. In this study, we measured the plasma concentration of NSE in both health controls plus early and late-stage patients and later investigated the potential of this marker to discriminate late stage from early-stage patients.

## Main text

### Materials and methods

#### Study design

We used a case–control study to compare plasma NSE levels between health controls, early and late-stage HAT patients and determine its ability to discriminate patients in either stage. We used *T. b. rhodesiense* archived plasma samples collected previously as described [13]. Routine diagnosis of suspects was done by microscopic examination blood films from finger prick blood [3]. For patients with positive blood smears, a lumbar puncture was performed and cerebrospinal fluid analysis for trypanosomes was performed after modified single centrifugation method [14] to stage patients. Neuron-specific enolase was assayed in plasma of both patients (early and late stage) and controls using sandwich ELISA at the Immunology laboratory, Makerere University.

#### Neuron-Specific Enolase immunoassay

Plasma NSE levels were measured using the human NSE ELISA kit (Elabscience^®^, USA) as described by the manufacturer. The standards were reconstituted with 1 mL of reference standard (150 ng/mL) and serially diluted to concentrations of; 75, 37.5, 18.75, 9.38, 4.69 and 2.34 ng/mL. ELISA plates were filled with 100 µl of standard, blank and sample and biotinylated detection antibody added, incubated and then washed. HRP conjugate working solution was added to each well and the sealed plate incubated for 30 min at 37 °C. Substrate solution was added to each well, covered with a new plate sealer and then incubated for 15 min at 37 °C. Finally, the stop solution was added to each well and optical density value for each well was determined at once using a micro-plate reader (Bio-Rad, USA) set at 450 nm.

#### Statistical analyses

All data were analyzed using GraphPad Prism version 8.0. A total of 66 HAT cases, N = 15 (early stage) and N = 51 (late stage) and 51 health controls were analyzed, power calculations for cases and controls are indicated in Additional file 1: Fig. S1 and for early and late-stage patients in Additional file 2. Fig. S2. Before analysis, deviation from normality was tested using D'Agostino–Pearson normality test. Since none of the data sets passed the normality test (P < 0.0001), comparison between cases and controls, early stage and late-stage patients were done with non-parametric tests. To determine the potential of NSE to be used as a stage biomarker, Receiver Operator Characteristic (ROC) curves were used to calculate the area under the ROC curve with sensitivity and specificity predictions for the different NSE concentration cut-offs recorded [15].

### RESULTS

#### Patient baseline characteristics

We retrieved plasma samples of 66 HAT patients and 51 healthy controls from the trypanosomiasis biobank at Makerere University. For all samples used, participants recruited passively at Lwala Hospital in North eastern Uganda between 2012 and 2014. As expected, late-stage cases were significantly more common (51 out of 66, (P < 0.0001) than early-stage patients. The sex-ratio (male: female) was 1:6 with a median age for HAT cases of 22 years (Table 1), sex for 7 patients could not be ascertained. The observed nonspecific signs of HAT and neurological signs are presented in Table 1.

**Table 1:** Patient’s baseline characteristics

Characteristic	Early stage	Late stage	P value
Disease stage	15	51 (77.2%)	< 0.0001*
Sex (male/female)	12/3	24/20	0.56
Age (median)	19	21	0.48
CSF parasitosis/mL	0	7 × 10^4^	< 0.000*
Fever	4	52 (92.8%)	0.09
Headache	6	45 (80.2%)	0.09
Hepatomegaly	0	2 (3.5%)	0.75
Splenomegaly	0	6 (10.7%)	0.14
Lymphadenopathy	2	8 (14.2%)	0.70
Gait abnormalities	0	11 (19.6%)	0.24
Tremors	0	9 (16.1%)	0.29
Urinary incontinence	0	7 (12.5%)	0.15

^*^Significantly higher in late-stage patients

#### Plasma Neuronal Specific Enolase (NSE) levels and stage progression

**Fig. 1:**
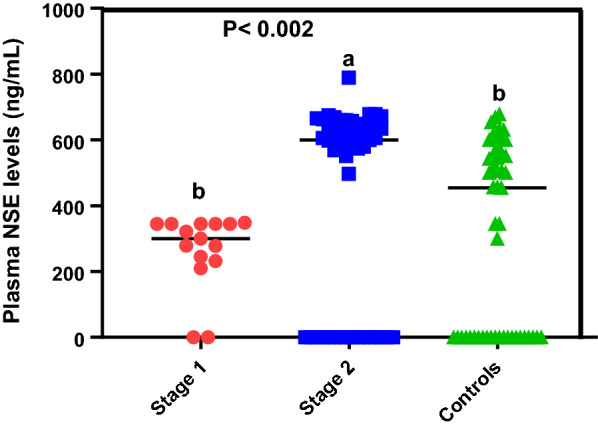
Plasma NSE levels in HAT patients and controls. Lowercase letters indicate significant differences between groups

In order to determine the differences between levels of NSE in the plasma of early and late-stage patients, a total of 66 HAT archived samples, N = 15 (early stage) and N = 51 (late stage) and 51 health controls were analyzed. The detection standards for the assays were 150, 75, 37.5, 18.75, 9.38, 4.68, and 2.34 ng/mL (Additional file 3: Fig. S3) with a detection limit of 1.85 ng/mL. Our result showed that median plasma NSE levels (ng/mL) significantly varied across study groups (Fig. 1, Kruskal Wallis test, P < 0.002). When NSE levels were compared across groups, results showed that median NSE levels were significantly higher (P = 0.009) among late-stage patients (599.8 ng/mL) when compared to controls (454 ng/mL). No significant differences (P > 0.9) in NSE levels were observed between early-stage patients and controls. When median NSE levels were compared between early and late-stage patients, results showed a significant (P < 0.02) upregulation among late-stage patients.

#### Neuronal Specific Enolase as a potential stage biomarker

**Fig. 2:**
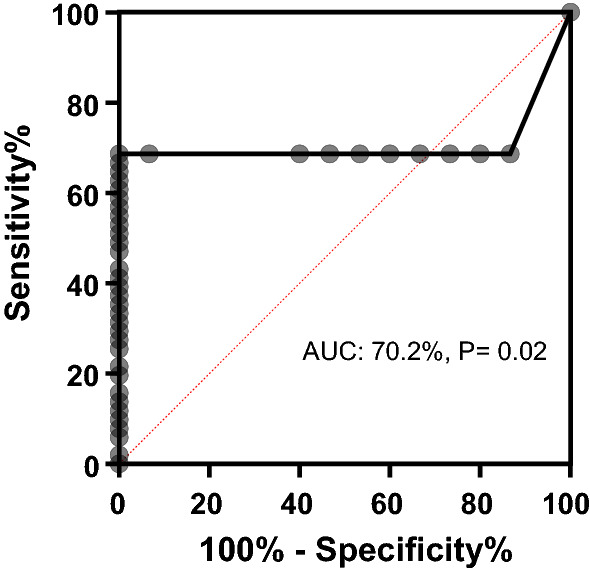
ROC curve analysis comparing potential of NSE in discriminating between early and late-stage HAT patients

In order to explore the possibility of using plasma NSE as a potential stage biomarker in discriminating between early and late-stage HAT patients, a Receiver Operator Characteristic (ROC) curve was used. Our results showed that NSE demonstrated an area under the curve (AUC) of 0.702 (95% CI 0.583–0.830, Fig. 2). A high staging accuracy for NSE was obtained by using a cutoff of > 346.5 ng/mL with a sensitivity of 68.6% (95% CI 55–79.7%) and a specificity of 93.3% (95% CI 70.2–99.7%).

### Discussion

With the observation that NSE is elevated in CSF and blood following traumatic brain injury, it was proposed as a potential biomarker for brain disorders. After neuronal damage, NSE leaks into the extracellular spaces and can be detected in CSF and blood [10]. In this study, we measured plasma levels of NSE in *T. b. rhodesiense* early and late-stage patients and made comparisons with health controls. Our results showed that median NSE levels were significantly higher among late-stage patients. Our results confirm earlier investigations by Sternberg and Mitchell who reported similar results while analyzing NSE levels among *T. b. rhodesiense* patients from Tororo and Serere in Eastern Uganda [12]. Indeed, several early studies have demonstrated elevated levels of NSE in CSF and blood samples from patients with brain disorders like, stroke [16–18], traumatic brain injury [19] and neuroendocrine cancers [20–22]. Those studies thus indicate that the plasma levels of NSE may indicate the extent of CNS damage. In HAT, Late-stage disease is similarly associated with remarkable CNS pathology in both murine models and human patients [13, 23, 24]. Indeed, in this study, no significant differences in NSE levels were observed between early-stage patients and controls.

Following observations that NSE is detectable in blood following brain injury, it has been proposed as a biomarker for a number of CNS inflammatory disorders. In our current study, we explored the potential of NSE in discriminating late stage from early-stage patients. The highest staging accuracy for NSE was obtained by using a cutoff of > 346.5 ng/mL with a sensitivity of 68.6% and a specificity of 93.3%. Although the area under the Receiver Operator Curve observed in this study was in line with what was reported previously by Sternberg and Mitchel [12], the sensitivity reported in our study remained low. However, for both studies the reported NSE sensitivity remain rather low to warranty any clinical use as a stage diagnostic biomarker. Furthermore, when Sternberg and Mitchel measured NSE levels in CSF of HAT patients, NSE levels remained low and concluded that the observed plasma NSE might have another source outside the CNS. Indeed, in small cell lung cancer (SCLC), NSE is of neuroendocrine origin and has been shown to be elevated in blood of SCLC patients albeit with inconclusive role about its applicability as a diagnostic marker [21, 25]. On the other hand, in patients with traumatic brain injury (TBI), serum NSE levels have been shown to be correlated with neurological outcomes, suggesting that NSE might be a diagnostic and prognostic biomarker for TBI albeit with inconclusive sensitivity [26].

Taken together, the use of NSE as potential stage specific biomarker poses apparent drawbacks. The inadequate sensitivity coupled with poor specificity means that NSE might not be used in isolation but rather as a panel of biomarkers [12, 27]. The other issue that might hinder the use of NSE as a blood based diagnostic biomarker is hemolysis that makes interpretation of elevated NSE levels challenging. Erythrocytes contain large amounts of NSE and thus tests measuring NSE would still relay on determination of the hemolysis index [28]. Although in our current study we did not measure hemoglobin levels, we followed similar sampling protocols as described by Sternberg and Mitchel [12] who demonstrated low hemoglobin levels with no association with plasma NSE levels.

### Conclusion

In this study, plasma NSE levels were elevated in late-stage *T. b. rhodesiense* HAT patients when compared with early-stage patients and health controls. Despite the observed NSE elevation in late-stage patients, the sensitivity of plasma NSE in discriminating between early and late-stage patients was inadequate. However, it is possible that the sensitivity and specificity of NSE as a stage specific biomarker might be improved when used as a panel with other identified plasma biomarkers.

### Study limitation

Our study has a limitation emanating from the small sample size used. The reason for this is firstly, because of the low incidence in the number of new *T. b. rhodesiense* reported cases. Secondly, because of the acuteness of the disease, majority of patients report to hospital when already in late stage and thus limiting the number of early-stage cases to making meaningful comparisons.

## Supplementary Information

**Additional file 1: Figure S1.** Power calculation for early and late-stage patients.

**Additional file 2: Figure S2**. Power calculation for cases and controls.

**Additional file 3: Figure S3.** Standard curve for NSE.

## Data Availability

The datasets used and/or analysed during the current study are available from the corresponding author on reasonable request.

## Abbreviations

AUC: Area under the ROC curve
CNS: Central nervous system
CSF: Cerebrospinal fluid
HAT: Human African trypanosomiasis
NSE: Neuronal Specific Enolase
ROC: Receiver Operator Characteristic
CT: Computed tomography
