# Supplementary material for: Plasma Neuron-Specific Enolase is not a reliable biomarker for staging Trypanosoma brucei rhodesiense sleeping sickness patients
Source: BMC Res Notes. 2022 Mar 7;15:97. doi: 10.1186/s13104-022-05981-w (PMC8900431; doi:10.1186/s13104-022-05981-w)
Supplement: Supplementary file 2 [file 13104_2022_5981_MOESM2_ESM.docx]

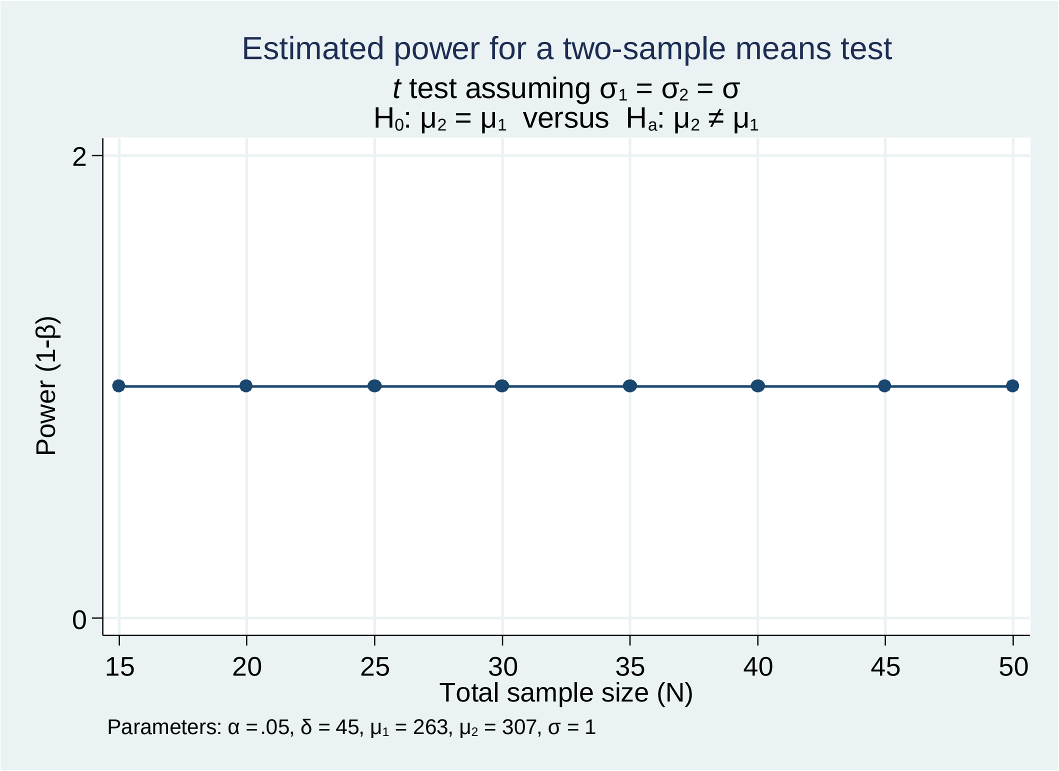
Estimated power for a two-sample means test
t test assuming σ1 = σ2 = σ
H0: μ2 = μ1 versus Ha: μ2 ≠ μ1
Power (1-β)
2
0
15
20
25
30
35
40
45
50
Total sample size (N)
Parameters: α = .05, δ = 45, μ1 = 263, μ2 = 307, σ = 1
