# Supplementary material for: Plasma Neuron-Specific Enolase is not a reliable biomarker for staging Trypanosoma brucei rhodesiense sleeping sickness patients
Source: BMC Res Notes. 2022 Mar 7;15:97. doi: 10.1186/s13104-022-05981-w (PMC8900431; doi:10.1186/s13104-022-05981-w)
Supplement: Supplementary file 3 [file 13104_2022_5981_MOESM3_ESM.docx]

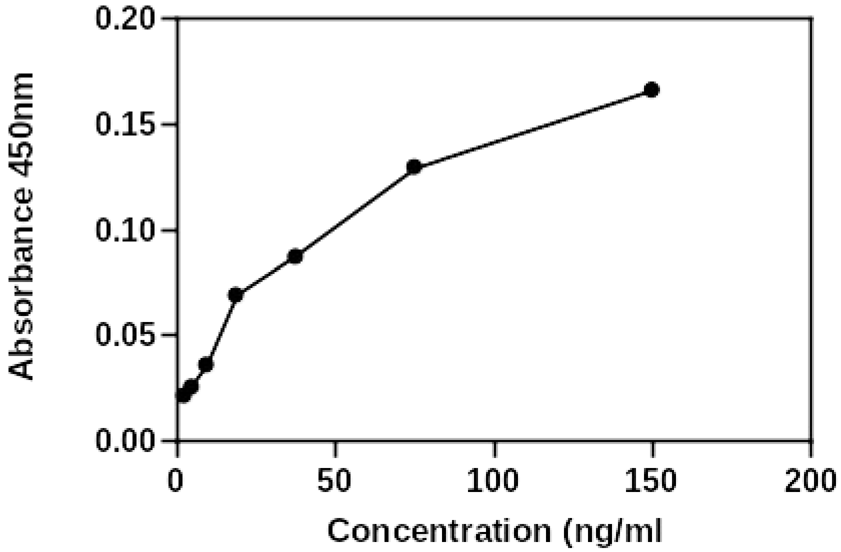

Absorbance 450nm
0.20
0.15
0.10
0.05
0.00
0
50
100
150
200
Concentration (ng/ml
